# Distributed Kalman Filtering Based on the Non-Repeated Diffusion Strategy

**DOI:** 10.3390/s20236923

**Published:** 2020-12-03

**Authors:** Xiaoyu Zhang, Yan Shen

**Affiliations:** 1 College of Artificial Intelligence, Nankai University, Tianjin 300350, China; zhangxiaoyu@nankai.edu.cn; 2 College of Intelligent Systems Science and Engineering, Harbin Engineering University, Harbin 150001, China

**Keywords:** distributed Kalman filter, diffusion strategy, sensor networks, data fusion

## Abstract

Estimation accuracy is the core performance index of sensor networks. In this study, a kind of distributed Kalman filter based on the non-repeated diffusion strategy is proposed in order to improve the estimation accuracy of sensor networks. The algorithm is applied to the state estimation of distributed sensor networks. In this sensor network, each node only exchanges information with adjacent nodes. Compared with existing diffusion-based distributed Kalman filters, the algorithm in this study improves the estimation accuracy of the networks. Meanwhile, a single-target tracking simulation is performed to analyze and verify the performance of the algorithm. Finally, by discussion, it is proved that the algorithm exhibits good all-round performance, not only regarding estimation accuracy.

## 1. Introduction

Sensor detection is undergoing a transition from an independent style to a cooperative style, and sensor networks have found increasing numbers of applications in areas such as in the Internet of Things [1], environmental monitoring [2], cooperative radar detection [3] and autonomous driving [4]. According to the types of communication topologies, sensor networks are classified into centralized networks and distributed networks [5]. A centralized network needs a stable central node with excellent communication performance, which is not yet realistic in large-scale networks. Therefore, a large number of researchers are focusing on distributed sensor networks [6,7].

Multiple nodes in a distributed sensor network can simultaneously detect and obtain target state information with noise. Each node communicates with its adjacent nodes. By using the distributed estimation algorithm, each node obtains a global and more accurate estimation than a single sensor. The distributed sensor network does not need to use a high-performance central node and is much more easily extended to a large-scale network. In short, this kind of sensor network has high stability and robustness and a low sensor cost [8]. Therefore, it is necessary to study distributed sensor networks. There are two main existing distributed Kalman filters, namely the consensus-based algorithm and diffusion-based algorithm.

The idea of consensus-based algorithms is to obtain the global average measure or the global average information vector and information matrix by performing the average consensus algorithm [9,10]. Olfatih–Saber first proposed a consensus-based distributed algorithm, called the Kalman consensus filter [11], which can obtain the mean value of global information vectors and the information matrix after infinite iterations and obtain the same global optimality estimations as centralized filters. In [12], the authors address the problem of the consensus of the information vector and information matrix, which requires infinite iterations to achieve the optimal estimation. To handle the correlation problem of estimations, in [13], the authors propose the information consensus filter (ICF), which fuses the prior information with low weights and the measured information of adjacent nodes. To handle local unobservability, a finite-time consensus-based distributed estimator is proposed in [14], which is based on the max-consensus technique. In [15,16], the authors propose a gossip-based algorithm that adopts a random communication strategy to achieve consensus. In contrast to consensus-based algorithms, a lower bandwidth can also guarantee the consensus of gossip-based algorithms with the cost of a slow convergence speed. Consensus-based algorithms have the same estimation accuracy as the centralized filter, whereas the global consensus is only achieved asymptotically.

Diffusion-based algorithms exchange intermediate estimations between the neighborhoods of each node and calculate the final estimation by a convex combination of information from neighbors. In [17,18], the authors obtain the sum of global measurement information in finite communication cycles through the diffusion of measurement information and propose a finite-time distributed Kalman filter (FT-DKF). In [19], the authors extend the algorithm proposed in [17] to cyclic graphs. The work presented in [20] was the first to propose a distributed estimation fusion filter based on diffusion. The accuracy of diffusion-based algorithms depends on the selection of convex combination coefficients. For this reason, in [21], the authors discuss the optimal selection of convex combination coefficients and formulate a constrained optimization problem. In [22], the authors propose the cost-effective diffusion Kalman filter (CE-DKF), which diffuses the information of state estimations and estimation covariances and improves the performance. The work presented in [23] was the first to apply the covariance intersection (CI) method to deal with the diffusion-based distributed Kalman filtering problem and address the problem of the system noise correlation between sensors. In [24], the authors use the local CI to improve local estimation performance. In [25], the authors proposed a new distributed Kalman filter that avoids the diffusion of raw data and maintains estimation accuracy by resorting to maximum posterior probability state estimation. In [26], the results of [25] are improved upon and the distributed hybrid information fusion (DHIF) algorithm is proposed. The partial diffusion Kalman filter (PDKF) is proposed for the state estimation of linear dynamic systems in [27]. By only sending partial estimation vectors at each iteration, the PDKF algorithm reduces the number of internode communication. In [28], the authors study the performance of PDKF for networks with noises. In summary, diffusion-based algorithms improve the consensus convergence speed, but the estimation accuracy is reduced at the same time.

From the above review, we can see that the diffusion-based algorithms have some advantages and some defects. Therefore, in this paper, we aim to design a kind of diffusion distributed Kalman filter for application to a sensor network. This sensor network is time-invariant and strongly connected. Each sensor node has its own information and the information from adjacent sensors. By using the distributed Kalman filter, the estimations of all nodes contain the global information and reflect the real state of the target after finite communication iterations.

First, a non-repeated diffusion strategy is introduced. The usual diffusion strategy is bi-directional; this means that the information diffuses back in some way. The non-repeated diffusion strategy involves removing the information that is received from a node before sending all messages to this node so that the information is not diffused back. This strategy makes information diffuse only in one direction, even in an undirected graph. Second, we apply the non-repeated diffusion strategy to the diffusion-based distributed Kalman filter and propose a new distributed Kalman filter in which the coefficients of convex combinations are obtained by CI. The introduction of the non-repeated diffusion strategy prevents sensors from fusing the same estimation repeatedly, which may lead to some estimations being too highly weighted and the final estimation results to be biased toward these estimations. Compared with existing distributed Kalman filters, the algorithm in this study has a lower error of estimations. Besides, we show that the algorithm also has good performance in other aspects, including communication bandwidth requirements, communication frequency requirements, applicability to different topologies and robustness to local unobservability. A trace simulation example is provided to verify the performance of the distributed Kalman filter based on the non-repeated diffusion strategy.

The rest of the paper is organized as follows. In Section 2, the system model and a diffusion distributed Kalman filter based on the non-repeated diffusion strategy are introduced. The result of the algorithm is shown in Section 3, which verifies the effectiveness of the algorithm. Section 4 discusses this result and describes the application scenarios of this algorithm. The conclusion of this paper is summarized in Section 5.

## 2. Diffusion Distributed Kalman Filtering Algorithms

### 2.1. System Model

In this study, the sensor network is represented by a graph G=(V,E), where V={1,2,3, …, N} is the set of sensor nodes (where *N* is the number of nodes in the sensor network and *E* is the set of communication channels (edges) between sensors). A graph is called an undirected graph if it consists of undirected edges. If an undirected edge (i,j)∈E exists, node *i* and node *j* are neighbors and can communicate with each other. We use $N_{i}$ to represent the set of adjacent nodes connected to the node *i*. When there is a path between each pair of different nodes *i* and *j*, the graph is called a connected graph. A path is called a cyclic path if this path starts from node A to node B and returns to node A through node C. If a graph is connected and has no cyclic path, it is called an acyclic graph (tree graph). We use *d* to represent the diameter of the graph *G*, which is the length of the longest path. $d_{ij}$ denotes the length of the shortest path between nodes *i* and *j*.

For the distributed estimation problem under this sensor network, we consider the following target state model

(1) x(t+1)=Ax(t)+Γω(t),

where $x\left(t\right)\in R^{n}$ is the state vector, $A\in R^{n\times n}$ is the state transition matrix, and $\Gamma \in R^{n\times h}$ is a noise coefficient matrix. $\omega \left(t\right)\in R^{h}$ is a system noise vector which is a zero-mean Gaussian white noise. The covariance matrix of ω(t) is Q>0. Each node of the sensor network can observe the target, and the measurement equation of each node is

(2) $$y_{i}\left(t\right)=H_{i}x\left(t\right)+v_{i}\left(t\right),$$

where $y_{i}\left(t\right)\in R^{m}$ is the measurement vector of sensor *i*, and $H\in R^{m\times n}$ is the observation matrix. $v_{i}\left(t\right)\in R^{m}$ is a measurement noise vector, which is a zero-mean Gaussian white noise. The covariance matrix of $v_{i}\left(t\right)$ is $R_{i}>0$. The covariance matrix of system noise ω(t) and measurement noise $v_{i}\left(t\right),\;\forall i\in V$ is

(3) $$E\left[\omega \left(t\right)v_{i}\left(t\right)\right]=O_{h\times m}.$$

The covariance matrix of measurement noise $v_{i}\left(t\right),\;\forall i\in V$ and measurement noise $v_{j}\left(t\right),$∀j∈V is

(4) $$E\left[v_{i}\left(t\right)v_{j}\left(t\right)\right]=O_{m\times m}.$$

To facilitate the expression, we introduce the following notations: $y_{i,t}$ is the measurement of target from the node *i* at time *t*, ${\hat{x}}_{i,t|t-1}$ is the state prediction at time *t* based on measurements by the sensor *i* up to time t−1, $P_{i,t|t-1}$ is an estimation error covariance matrix of ${\hat{x}}_{i,t|t-1}$. ${\hat{x}}_{i,t|t}$ and $P_{i,t|t}$ are the same as ${\hat{x}}_{i,t|t-1}$ and $P_{i,t|t-1}$.

### 2.2. A New Diffusion Distributed Kalman Filter

In this subsection, we propose a new diffusion distributed Kalman filter. First, we introduce the seminal diffusion distributed Kalman filter [20], which is summarized in Algorithm 1. ${\hat{x}}_{i,t|t}^{\mathrm{loc}}$ is the estimation with local information. $N_{i}+i$ is a set composed of set $N_{i}$ and node *i*, which contains node *i* and all adjacent nodes of node *i*.
**Algorithm 1:** The seminal diffusion distributed Kalman filter.  For the node i∈V and node $j\in N_{i}$,  Initialize with:    ${\hat{x}}_{i,0|0}=Ex_{0},$    $P_{i,0|0}=E\left[\left(x_{0}-Ex_{0}\right){\left(x_{0}-Ex_{0}\right)}^{T}\right];$  Local update:    ${\hat{x}}_{i,t|t-1}=A{\hat{x}}_{i,t-1|t-1}$,    $P_{i,t|t-1}=AP_{i,t-1|t-1}A^{T}+\Gamma Q\Gamma^{T}$,    ${\left(P_{i,t|t}\right)}^{-1}={\left(P_{i,t|t-1}\right)}^{-1}+H_{i}^{T}R_{i}^{-1}H_{i}$,    ${\hat{x}}_{i,t|t}^{\mathrm{loc}}={\hat{x}}_{i,t|t-1}+P_{i,t|t}H_{i}^{T}R_{i}^{-1}\left(y_{i,t}-H_{i}{\hat{x}}_{i,t|t-1}\right)$;  Communication and fusion update:    Send ${\hat{x}}_{j,t|t}^{\mathrm{loc}}$ to adjacent node *i*,    ${\hat{x}}_{i,t|t}=\sum_{k\in N_{i}+i}w_{k}{\hat{x}}_{k,t|t}^{\mathrm{loc}},$  where    $\sum_{k\in N_{i}+i}w_{k}=1,\;0\leq w_{k}\leq 1$.

The algorithm fuses the estimation of adjacent nodes, increases the information exchange and improves the estimation accuracy compared with the Kalman filter of a single sensor. The shortcoming of this algorithm is that it does not deal with the estimation error covariance. Therefore, $P_{i,t-1|t-1}$ in Algorithm 1 is not the accurate estimation error covariance of ${\hat{x}}_{i,t-1|t-1}$ in Algorithm 1, and the estimation accuracy of Algorithm 1 is reduced. In this work, we diffuse and fuse the estimation error covariance to optimize Algorithm 1.

In general, due to the process noise and complex sensor interactions, the cross-covariance between different estimations is unknown. In the case of unknown cross-covariance, we can use CI [25], the ellipsoidal intersection (EI) method [29] and the information sharing principle [30], etc. The information sharing principle requires the distribution of the covariance of global estimation to each node according to a certain proportion, which leads to a great burden of communication. From Algorithm 1, we know that the diffusion-based distributed Kalman filtering algorithm is a convex combination of estimations of adjacent nodes. Similarly, the EI method and CI method obtain more accurate estimations by adjusting the convex combination coefficients of multiple estimates. From this perspective, we can apply the EI method and CI method to extend the diffusion-based distributed filtering algorithm. However, the EI method has some disadvantages compared with the CI method. First, the EI method can provide a more accurate estimation, but its result does not result in consistent estimation. Second, the choice of whether to use EI method is an engineering problem. Third, the calculation process of the EI method is complex, which results in it being inconvenient to describe the algorithm proposed in this paper. Therefore, we use the CI method to fuse estimations, as described in Algorithm 2.

In Algorithm 2, $P_{i,t|t}^{\mathrm{loc}}$ is the estimation error covariance of ${\hat{x}}_{i,t|t}^{\mathrm{loc}}$ and $w_{i},\;i\in V$ are weight coefficients of CI that minimize the following equation:

(5) $$\min_{w_{i}\geq 0,\sum_{i\in V}w_{i}=1}[\mathrm{tr}(\sum_{i\in V}w_{i}P_{i,t|t}^{-1})]$$

with tr(·) being the trace function. Equation (5) is a nonlinear optimization problem whose computational cost is usually expensive and unaffordable for sensors. The weight can be set as the mean to reduce computational cost.
**Algorithm 2:** A centralized Kalman filter based on the covariance intersection (CI) method.  For the node i∈V,  Initialize with:    ${\hat{x}}_{i,0|0}^{\mathrm{loc}}=Ex_{0},$    $P_{i,0|0}^{\mathrm{loc}}=E\left[\left(x_{0}-Ex_{0}\right){\left(x_{0}-Ex_{0}\right)}^{T}\right];$  Local update:    $P_{i,t|t-1}^{\mathrm{loc}}=AP_{i,t-1|t-1}^{\mathrm{loc}}A^{T}+\Gamma Q\Gamma^{T}$,    ${\hat{x}}_{i,t|t-1}^{\mathrm{loc}}=A{\hat{x}}_{i,t-1|t-1}^{\mathrm{loc}}$,    ${\hat{x}}_{i,t|t}^{\mathrm{loc}}={\hat{x}}_{i,t|t-1}^{\mathrm{loc}}+H_{i}^{T}R_{i}^{-1}\left(y_{i,t-1}-H_{i}{\hat{x}}_{i,t|t-1}^{\mathrm{loc}}\right)$,    ${\left(P_{i,t|t}^{\mathrm{loc}}\right)}^{-1}={\left(P_{i,t|t-1}^{\mathrm{loc}}\right)}^{-1}+H_{i}^{T}R_{i}^{-1}H_{i};$  Fusion update:    $P_{t|t}^{-1}=\sum_{i\in V}w_{i}{\left(P_{i,t|t}^{\mathrm{loc}}\right)}^{-1}$,    ${\hat{x}}_{t|t}=P_{t|t}\sum_{i\in V}w_{i}{\left(P_{i,t|t}^{\mathrm{loc}}\right)}^{-1}{\hat{x}}_{i,t|t}^{\mathrm{loc}}$,  where $w_{j}$ is calculated by Equation (5).

The error covariance of estimation calculated by CI is approximate, and so the result of Algorithm 2 is also approximate, whereas CI provides a conservative result which guarantees that the accuracy in a specific direction is not lower than that of any node in this direction. The estimation of Algorithm 2 and the optimal estimation satisfy the following equation:

(6) $${\hat{x}}_{t|t}\approx E\left(x_{t}|y_{i,0},y_{i,1},\;\ldots ,\;y_{i,t},i\in V\right).$$

Applying Algorithm 2 to Algorithm 1, we obtain a diffusion distributed Kalman filter that fuses estimation and its error covariance concurrently, which is presented in Algorithm 3.
**Algorithm 3:** A diffusion distributed Kalman filter with the CI method.  For the node i∈V and node $j\in N_{i}$,  Initialize with:    ${\hat{x}}_{i,0|0}=Ex_{0}$,    $P_{i,0|0}=E\left[\left(x_{0}-Ex_{0}\right){\left(x_{0}-Ex_{0}\right)}^{T}\right];$  Local update:    ${\hat{x}}_{i,t|t-1}=A{\hat{x}}_{i,t-1|t-1}$,    $P_{i,t|t-1}=AP_{i,t-1|t-1}A^{T}+\Gamma Q\Gamma^{T}$,    ${\left(P_{i,t|t}^{\mathrm{loc}}\right)}^{-1}={\left(P_{i,t|t-1}\right)}^{-1}+H_{i}^{T}R_{i}^{-1}H_{i}$,    ${\hat{x}}_{i,t|t}^{\mathrm{loc}}={\hat{x}}_{i,t|t-1}+P_{i,t|t}H_{i}^{T}R_{i}^{-1}\left(y_{i,t}-H_{i}{\hat{x}}_{i,t|t-1}\right)$;  Communication and fusion update:    Send ${\hat{x}}_{j,t|t}^{\mathrm{loc}}$ and $P_{j,t|t}^{\mathrm{loc}}$ to adjacent node *i*,    $P_{i,t|t}^{-1}=\sum_{k\in N_{i}+i}w_{k}{\left(P_{k,t|t}^{\mathrm{loc}}\right)}^{-1}$,    ${\hat{x}}_{i,t|t}=P_{i,t|t}\left[\sum_{k\in N_{i}+i}w_{k}{\left(P_{k,t|t}^{\mathrm{loc}}\right)}^{-1}{\hat{x}}_{k,t|t}^{\mathrm{loc}}\right],$        
(7)
  where $w_{k}$ is calculated by Equation (5).

In Algorithm 3, ${\hat{x}}_{i,t|t}^{\mathrm{loc}}$ is calculated from ${\hat{x}}_{i,t-1|t-1}$ by local update. ${\hat{x}}_{i,t-1|t-1}$ fuses information of ${\hat{x}}_{j,t-1|t-1}^{\mathrm{loc}}$. Therefore, ${\hat{x}}_{i,t|t}^{\mathrm{loc}}$ also contains information of ${\hat{x}}_{j,t-1|t-1}^{\mathrm{loc}}$ and ${\hat{x}}_{i,t|t}^{\mathrm{loc}}$ is correlative to ${\hat{x}}_{j,t-1|t-1}^{\mathrm{loc}}$. Since ${\hat{x}}_{j,t|t}^{\mathrm{loc}}$ is also correlative to ${\hat{x}}_{j,t-1|t-1}^{\mathrm{loc}}$, ${\hat{x}}_{i,t|t}^{\mathrm{loc}}$ is correlative to ${\hat{x}}_{j,t|t}^{\mathrm{loc}}$. In Equation (7), Algorithm 3 fuses ${\hat{x}}_{i,t|t}^{\mathrm{loc}}$ and ${\hat{x}}_{j,t|t}^{\mathrm{loc}}$ and omits their correlation. In order to improve the accuracy of Algorithm 3, we separate diffusion update from local update, and propose a diffusion distributed Kalman filter based on CI in Algorithm 4.
**Algorithm 4:** A diffusion distributed Kalman filter separating diffusion update.  For the node i∈V and node $j\in N_{i}$,  Initialize with:    ${\hat{x}}_{i,0|0}^{\mathrm{loc}}=Ex_{0}$,    $P_{i,0|0}^{\mathrm{loc}}=E\left[\left(x_{0}-Ex_{0}\right){\left(x_{0}-Ex_{0}\right)}^{T}\right]$,    ${\hat{x}}_{i,0|0}=Ex_{0}$,    $P_{i,0|0}=E\left[\left(x_{0}-Ex_{0}\right){\left(x_{0}-Ex_{0}\right)}^{T}\right];$  Local update:    ${\hat{x}}_{i,t|t-1}^{\mathrm{loc}}=A{\hat{x}}_{i,t-1|t-1}^{\mathrm{loc}}$,    $P_{i,t|t-1}^{\mathrm{loc}}=AP_{i,t-1|t-1}^{\mathrm{loc}}A^{T}+\Gamma Q\Gamma^{T}$,    ${\left(P_{i,t|t}^{\mathrm{loc}}\right)}^{-1}={\left(P_{i,t|t-1}^{\mathrm{loc}}\right)}^{-1}+H_{i}^{T}R_{i}^{-1}H_{i}$,    ${\hat{x}}_{i,t|t}^{\mathrm{loc}}={\hat{x}}_{i,t|t-1}^{\mathrm{loc}}+P_{i,t|t}^{\mathrm{loc}}H_{i}^{T}R_{i}^{-1}\left(y_{i,t}-H_{i}{\hat{x}}_{i,t|t-1}^{\mathrm{loc}}\right)$;  Diffusion incremental Update:    ${\hat{x}}_{i,t|t-1}=A{\hat{x}}_{i,t-1|t-1}$,    $P_{i,t|t-1}=AP_{i,t-1|t-1}A^{T}+\Gamma Q\Gamma^{T}$,    ${\left(P_{i,t|t}^{\mathrm{diffusion}}\right)}^{-1}=P_{i,t|t-1}^{-1}+H_{i}^{T}R_{i}^{-1}H_{i}$,    ${\hat{x}}_{i,t|t}^{\mathrm{diffusion}}={\hat{x}}_{i,t|t-1}+P_{i,t|t}^{\mathrm{diffusion}}H_{i}^{T}R_{i}^{-1}\left(y_{i,t}-H_{i}{\hat{x}}_{i,t|t-1}\right)$;  Communication and fusion update:    Send ${\hat{x}}_{j,t|t}^{\mathrm{diffusion}}$ and $P_{j,t|t}^{\mathrm{diffusion}}$ to adjacent node *i*,    $P_{i,t|t}^{-1}=\sum_{j\in N_{i}}w_{j}{\left(P_{i,t|t}^{\mathrm{diffusion}}\right)}^{-1}+w_{i}{\left(P_{i,t|t}^{\mathrm{loc}}\right)}^{-1}$,    ${\hat{x}}_{i,t|t}=P_{i,t|t}\left[\sum_{j\in N_{i}}w_{j}{\left(P_{i,t|t}^{\mathrm{diffusion}}\right)}^{-1}{\hat{x}}_{j,t|t}^{\mathrm{diffusion}}+w_{i}{\left(P_{i,t|t}^{\mathrm{loc}}\right)}^{-1}{\hat{x}}_{i,t|t}^{\mathrm{loc}}\right],$  where $w_{j}$ and $w_{i}$ is calculated by Equation (5).

In Algorithm 4, each node requires a local update and diffusion incremental update. The results ${\hat{x}}_{i,t|t}^{\mathrm{diffusion}}$ and $P_{i,t|t}^{\mathrm{diffusion}}$ of the incremental diffusion update are diffused to adjacent nodes. The results ${\hat{x}}_{i,t|t}$ and $P_{i,t|t}^{\mathrm{loc}}$ of the local update are used to fuse with the received information. Compared with Algorithm 3, Algorithm 4 uses the local information to fuse with the received information in the fusion process instead of using the fusion results of the last moment to fuse with the received information; this solves the correlation problem caused by fusing the received information of the last moment with the received information at the current moment in Algorithm 3 at every moment.

### 2.3. Distributed Kalman Filter Based on the Non-Repeated Diffusion Strategy

In this subsection, we proposed a distributed Kalman filter based on the non-repeated diffusion strategy and analyze its performance. First, we introduce the non-repeated diffusion strategy in Algorithm 5. Each node in Algorithm 5 has local information $I_{i,t}^{\mathrm{loc}}$ at each moment. $I_{i,t}$ is all of the information of node *i* at time *t* and is calculated by Equation (9). $I_{i\to i_{1},t}$ is the information that is sent from node *i* to node $i_{1}$ at time *t* and is calculated by Equation (8). In this strategy, the diffusion information for each adjacent node is calculated. The diffusion information $I_{i\to i_{1},t}$ is calculated by subtracting the received information from node $i_{1}$ at the last moment from all information at the last moment. The strategy avoids the sent information from a node returning to this node again.
**Algorithm 5:** The non-repeated diffusion strategy.  For the node i∈V and node $i_{1}\in N_{i}$,  Initialize information of node *i* at time *t* with $I_{i,t}^{\mathrm{loc}}$;  Initialize with:    $I_{i_{1}\to i,0}=0$;  Diffusion update:    $I_{i\to i_{1},t}=\sum_{i_{2}\in N_{i}-i_{1}}I_{i_{2}\to i,t-1}+I_{i,t}^{\mathrm{loc}}$;    (8)  Communication and fusion information:    Send $I_{i_{1}\to i,t}$ to adjacent node *i*,    $I_{i,t}=\sum_{i_{1}\in N_{i}}I_{i_{1}\to i,t}+I_{i,t}^{\mathrm{loc}}.$    (9)

**Assumption** **1.** *The sensor network is an acyclic graph.*

**Theorem** **1.** *Let Assumption 1 hold. Running Algorithm 5, no node of the sensor network will ever receive its own information.*

**Proof** **of** **Theorem** **1.** There is a node *i* in an acyclic graph. Substituting Equation (8) into Equation (9), we obtain

(10) $$I_{i,t}=\sum_{i_{1}\in N_{i}}\left(\sum_{i_{2}\in N_{i_{1}}-i}I_{i_{2}\to i_{1},t-1}+I_{i_{1},t}^{\mathrm{loc}}\right)+I_{i,t}^{\mathrm{loc}}.$$

Substituting Equation (8) into Equation (10) many times, all information of node *i* at time *t* can be presented as

(11) $$I_{i,t}=\sum_{i_{1}\in N_{i}}\left(\sum_{i_{2}\in N_{i_{1}}-i}\cdots (\sum_{i_{n}\in N_{i_{n-1}}-i_{n-2}}I_{i_{n}\to i_{n-1},t-\left(n-1\right)}+I_{i_{n-1},t-\left(n-2\right)}^{\mathrm{loc}})\cdots +I_{i_{1},t}^{\mathrm{loc}}\right)+I_{i,t}^{\mathrm{loc}},$$

where $n\in N^{*}$. The sets of adjacent nodes of any two points in an acyclic graph do not produce an intersection set (except for each other); otherwise, there will be a cyclic path through the intersection set. Thus, there is no intersection set among $N_{i_{n-1}}-i_{n-2},n\in N^{*}$ and the element of $N_{i_{n-1}}-i_{n-2}$ will constantly decrease as *n* increases. There is an *m* that means that $N_{i_{n-1}}-i_{n-2}=\emptyset ,n\geq m$. At this moment, all information of node *i* is reduced as

(12) $$I_{i,t}=I_{i,t}^{\mathrm{loc}}+\sum_{i_{1}\in N_{i}}I_{i_{1},t}^{\mathrm{loc}}+\sum_{i_{2}\in N_{i_{1}}}I_{i_{2},t}^{\mathrm{loc}}+\cdots +\sum_{i_{m}\in N_{i_{m-1}}}I_{i_{m},t-\left(m-1\right)}^{\mathrm{loc}},$$

where $I_{i,t}^{\mathrm{loc}}$ is the information that node *i* already has. Therefore, the node *i* never receives its own information from other nodes. □

We introduce the non-repeated diffusion strategy into Algorithm 4 and propose a new diffusion distributed Kalman filter, which is the core of this paper and is presented in Algorithm 6. In Algorithm 6, ${\hat{x}}_{i\to j,t|t}$ and $P_{i\to j,t|t}$ are the estimation and its error covariance diffused from node *i* to *j*.
**Algorithm 6:** A distributed Kalman filter based on the non-repeated diffusion strategy.  For the node i∈V and node $j\in N_{i}$.  Initialize with:    ${\hat{x}}_{i,0|0}^{\mathrm{loc}}=Ex_{0},P_{i,0|0}^{\mathrm{loc}}=E\left[\left(x_{0}-Ex_{0}\right){\left(x_{0}-Ex_{0}\right)}^{T}\right]$,    ${\hat{x}}_{j\to i,0|0}=0,P_{j\to i,0|0}=E\left[\left(x_{0}-Ex_{0}\right){\left(x_{0}-Ex_{0}\right)}^{T}\right]$;  Local update:    ${\hat{x}}_{i,t|t-1}^{\mathrm{loc}}=A{\hat{x}}_{i,t-1|t-1}^{\mathrm{loc}}$,    $P_{i,t|t-1}^{\mathrm{loc}}=AP_{i,t-1|t-1}^{\mathrm{loc}}A^{T}+\Gamma Q\Gamma^{T}$,    ${\left(P_{i,t|t}^{\mathrm{loc}}\right)}^{-1}={\left(P_{i,t|t-1}^{\mathrm{loc}}\right)}^{-1}+H_{i}^{T}R_{i}^{-1}H_{i}$,    ${\hat{x}}_{i,t|t}^{\mathrm{loc}}={\hat{x}}_{i,t|t-1}^{\mathrm{loc}}+P_{i,t|t}^{\mathrm{loc}}H_{i}^{T}R_{i}^{-1}\left(y_{i,t}-H_{i}{\hat{x}}_{i,t|t-1}^{\mathrm{loc}}\right)$;  Diffusion incremental update:    $P_{i\to j,t}^{-1}=\sum_{g\in N_{i}-j}w_{j,g\to i}P_{g\to i,t-1|t-1}^{-1}+w_{j,i\to i}{\left(P_{i,t-1|t-1}^{\mathrm{loc}}\right)}^{-1},$    (13)    ${\hat{x}}_{i\to j,t}=P_{i\to j,t}\left[\sum_{g\in N_{i}-j}w_{j,g\to i}P_{g\to i,t|t}^{-1}{\hat{x}}_{g\to i,t-1|t-1}+w_{j,i\to i}{\left(P_{i,t-1|t-1}^{\mathrm{loc}}\right)}^{-1}{\hat{x}}_{i,t-1|t-1}^{\mathrm{loc}}\right],$    (14)    ${\hat{x}}_{i\to j,t|t-1}=A{\hat{x}}_{i\to j,t}$,    $P_{i\to j,t|t-1}=AP_{i\to j,t}A^{T}+\Gamma Q\Gamma^{T}$,    $P_{i\to j,t|t}^{-1}=P_{i\to j,t|t-1}^{-1}+H_{i}^{T}R_{i}^{-1}H_{i}$,    ${\hat{x}}_{i\to j,t|t}={\hat{x}}_{i\to j,t|t-1}+P_{i\to j,t|t}H_{i}^{T}R_{i}^{-1}\left(y_{i,t}-H_{i}{\hat{x}}_{i\to j,t|t-1}\right)$;  Communication and fusion update:    Send ${\hat{x}}_{j\to i,t|t}$ and $P_{j\to i,t|t}$ to adjacent node *i*,    $P_{i,t|t}^{-1}=\sum_{j\in N_{i}}w_{j\to i}P_{j\to i,t|t}^{-1}+w_{i\to i}{\left(P_{i,t|t}^{\mathrm{loc}}\right)}^{-1},$    ${\hat{x}}_{i,t|t}=P_{i,t|t}\left[\sum_{j\in N_{i}}w_{j\to i}P_{j\to i,t|t}^{-1}{\hat{x}}_{j\to i,t|t}+w_{i\to i}{\left(P_{i,t|t}^{\mathrm{loc}}\right)}^{-1}{\hat{x}}_{i,t|t}^{\mathrm{loc}}\right],$  where $w_{j}$ is calculated by Equation (19).

**Theorem** **2.** *Let Assumption 1 hold. The estimations calculated by Algorithm 6 fuse the global information and reflect the real state of the target after a finite number of communications.*

**Proof** **of** **Theorem** **2.** The step of the fusion update in Algorithm 4 combines information from adjacent nodes of node *i* linearly. In the step of the diffusion update, all information of node *i* is diffused to all adjacent nodes. Therefore, adjacent nodes of node *i* can receive the information from the other adjacent nodes of node *i*. In every communication, the information of each node is diffused to the next node. Algorithm 6 adds two steps—Equations (13) and (14)—which are derived from the non-repeated diffusion strategy of Algorithm 5. From Theorem 1, we know that Algorithm 6 realizes the prerequisite that each node never receives its own information. Since the information is diffused to the next node and each node does not receive its own information, the information is always diffused in one direction. Each node can receive the information from other nodes besides its neighbors. The amount of received information depends on the number of other nodes within a certain distance. Due to applying CI to the fusing of the information, a conservative estimation is obtained that is the same as Equation (6). The estimation of node *i* is

(15) $${\hat{x}}_{i,t|t}\approx E\left(x_{t}|y_{l,1},\;\ldots ,\;y_{l,t_{il}},l\in V^{\prime }\right),$$

where

(16) $$t_{il}=t-d_{il}+1,$$

and $V^{\prime }$ is a set of nodes, whose measures can be received by node *i*. $\left\{y_{l,1},\;\ldots ,\;y_{l,t_{il}},l\in V^{\prime }\right\}$ represents the measurements of nodes in $V^{\prime }$, and $t_{il}$ denotes the maximum moment before which the measurement information from node *l* can be received by node *i*.

Assume that the two nodes with the longest distance in the sensor network are node *i* and node *j* ($d_{ij}=d$); with *d* communications after time $t_{1}$ (at time $t=t_{1}+d-1$), we obtain

(17) $$t_{il}=t_{1}+d-d_{il},\forall l\in V.$$

For node *j*, there is

(18) $$t_{ij}=t_{1}.$$

At this moment, node *i* receives the measure $y_{j,t_{1}}$. Each node can receive the estimates and covariances of all nodes in the sensor network because the two nodes with the longest distance can communicate. Due to the fusing of the estimations and covariances of all nodes by CI, each node obtains an estimation that includes the global information and reflects the real state of target after *d* communications. □

$w_{j,g\to i},w_{j,i\to i},w_{j\to i}$ and $w_{i\to i}$ in Algorithm 6 are the weight coefficients of CI, which minimize the following equation (take $w_{j\to i},\;w_{i\to i}$ as an example):

(19) $$\min_{w_{j\to i}\geq 0,w_{i\to i}\geq 0,\sum_{j\in N_{i}}w_{j\to i}+w_{i\to i}=1}[\mathrm{tr}(\sum_{j\in N_{i}}w_{j\to i}\xi_{j\to i}+w_{i\to i}{\left(P_{i,t-1|t-1}^{\mathrm{loc}}\right)}^{-1})].$$

Equation (19) is a nonlinear optimization problem, as with Equation (5), whose computational cost is also expensive and unaffordable for sensors. The weight can be set as the mean to reduce computational cost.

## 3. Results

In this section, an example is simulated to show the efficiency of Algorithm 6, and the comparisons with CE-DKF [22] and DHIF [26] are also shown. CE-DKF and DHIF are two existing prominent diffusion-based distributed filter algorithms.

**Figure 1:**
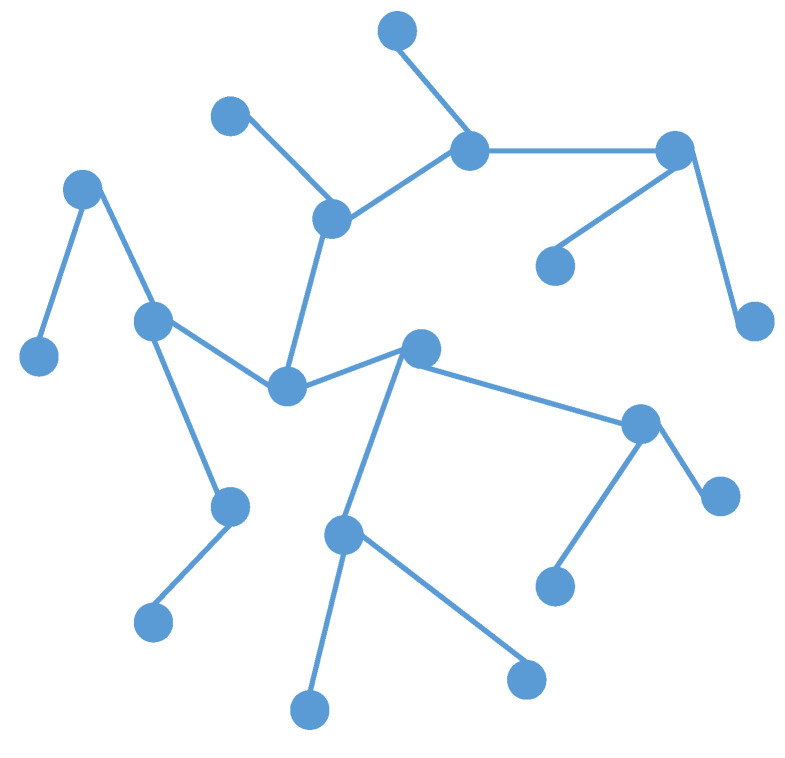
A sensor network with 20 nodes.

Specifically, we consider a sensor network of 20 nodes whose topological structure is shown in Figure 1. Each node can detect the target and communicate bi-directionally with its adjacent nodes. The linear dynamical system of Equation (1) is considered, where the state vector $x_{t}={\left[\alpha_{t}\;\beta_{t}\;{\dot{\alpha }}_{t}\;{\dot{\beta }}_{t}\right]}^{T}$ consists of position $\alpha_{t},\beta_{t}$ and speed ${\dot{\alpha }}_{t},{\dot{\beta }}_{t}$ from horizontal and vertical directions, respectively. The unit is meters. The initial state is ${\left[0\;0\;20\;20\right]}^{T}$. The acceleration of the target is modeled as the system noise $\omega \left(t\right)={\left[{\ddot{\alpha }}_{t}\;{\ddot{\beta }}_{t}\right]}^{T}$ whose mean is ${\left[0\;0\right]}^{T}$ and variance is *Q*. Here, we consider the case where *Q* is equal to

$$Q_{1}={\left[\begin{array}{cc} 10 & 0\\ 0 & 10 \end{array}\right]}^{T}$$

or

$$Q_{2}={\left[\begin{array}{cc} 100 & 0\\ 0 & 100 \end{array}\right]}^{T}.$$

The state transition matrix is

$$A=\left[\begin{array}{cccc} 1 & 0 & 1 & 0\\ 0 & 1 & 0 & 1\\ 0 & 0 & 1 & 0\\ 0 & 0 & 0 & 1 \end{array}\right],$$

and the noise coefficient matrix is

$$\Gamma =\left[\begin{array}{cc} 0.5 & 0\\ 0 & 0.5\\ 1 & 0\\ 0 & 1 \end{array}\right].$$

The sensor measurement model is Equation (2), where

$$H=\left[\begin{array}{cccc} 1 & 0 & 0 & 0\\ 0 & 1 & 0 & 0\\ 0 & 0 & 1 & 0\\ 0 & 0 & 0 & 1 \end{array}\right],$$

the mean of $v_{i}\left(t\right)$ is ${\left[0\;0\;0\;0\right]}^{T}$, and the variance of $v_{i}\left(t\right)$ is

$$R_{i}={\left[\begin{array}{cccc} 5 & 0 & 0 & 0\\ 0 & 5 & 0 & 0\\ 0 & 0 & 1 & 0\\ 0 & 0 & 0 & 1 \end{array}\right]}^{T},\;\forall i\in V.$$

Next, we perform a simulation 2000 times to compare Algorithm 6 with other distributed filtering algorithms. The fusion weights of both Algorithm 6 and DHIF are mean values.

**Figure 2:**
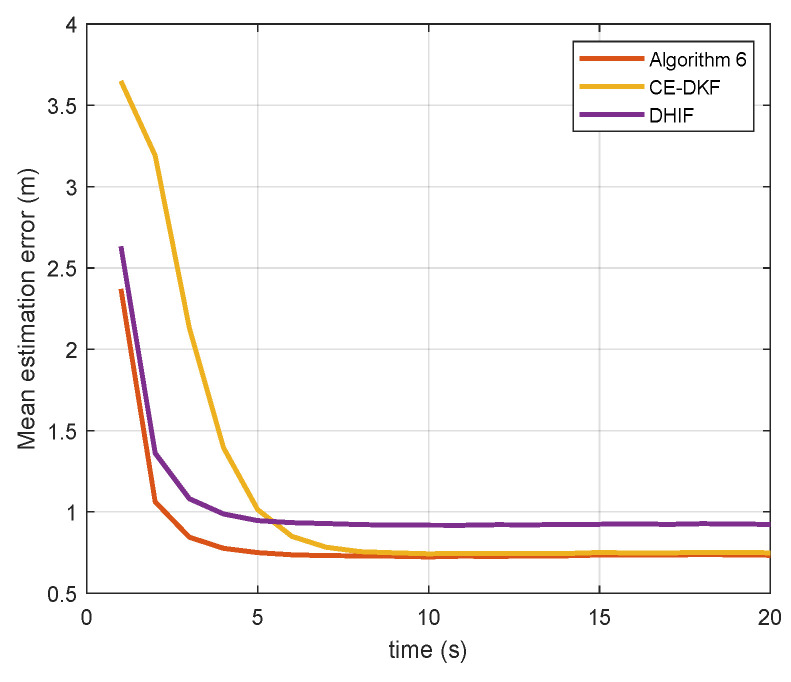
Mean estimation errors of different estimation algorithms ($Q=Q_{1}$). CE-DKF: cost-effective diffusion Kalman filter; DHIF: distributed hybrid information fusion.

**Figure 3:**
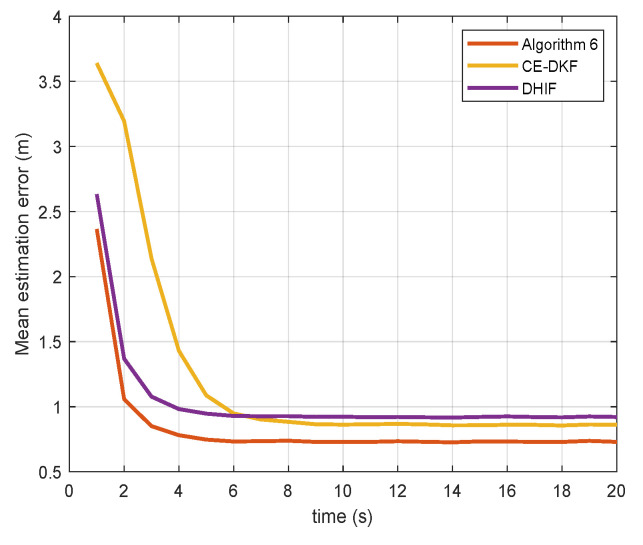
Mean estimation errors of different estimation algorithms ($Q=Q_{2}$).

Figure 2 and Figure 3 show the estimation error of the algorithms for state $\alpha_{t}$. The equation to calculate the error is

(20) $$\varepsilon =\sqrt{\frac{1}{N}\sum_{i\in V}{\left(\alpha_{i,t|t}-\alpha_{t}\right)}^{2}}.$$

The data of Figure 2 and Figure 3 are collated in Table 1.

The error reduction of Algorithm 6 is the percentage of error that Algorithm 6 reduces compared to other algorithms. The error convergence time is the earliest time when the error reaches a steady state. From Table 1, we see that Algorithm 6 reduces the estimation error by up to 20.97% and reaches a steady state faster than CE-DKF and DHIF. For the case in which the noise covariance matrix *Q* is large, the performance of Algorithm 6 is more prominent. In the case of a small *Q*, Algorithm 6 only reduces the error by 2.34% compared with CE-DKF. Because the maneuverability of target is small in this case, the advantages of Algorithm 6 are not obvious.

**Figure 4:**
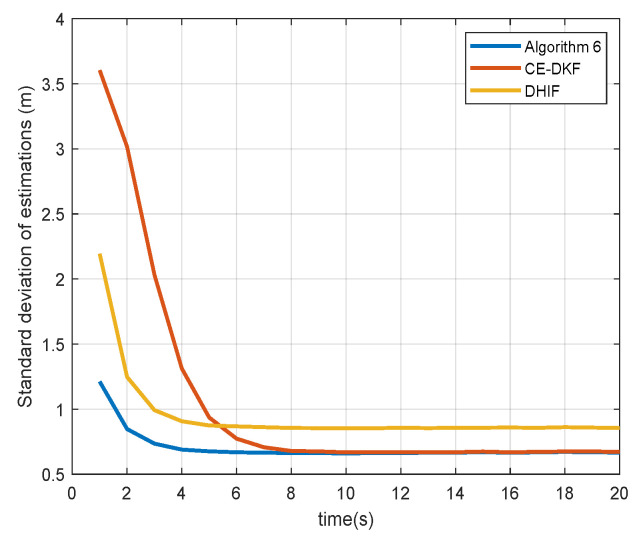
Standard deviation of estimations ($Q=Q_{1}$).

**Figure 5:**
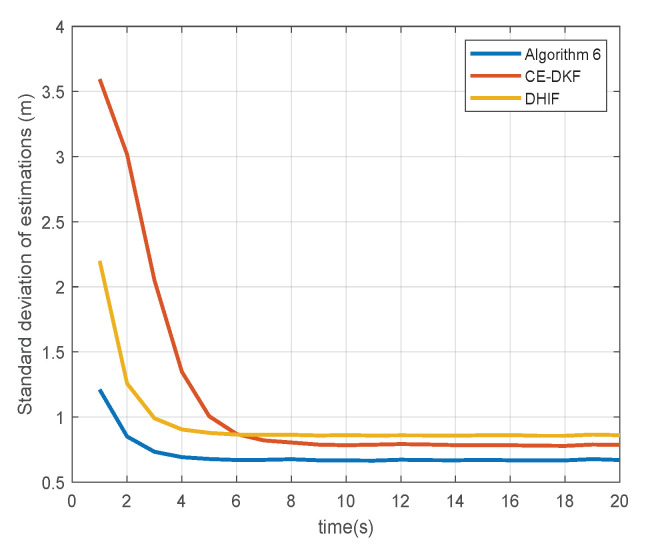
Standard deviation of estimations ($Q=Q_{2}$).

Figure 4 and Figure 5 show the standard deviations (SDs) of the estimations of sensors for state $\alpha_{t}$, which represents the degree of consensus of estimations from all nodes in the sensor network and is calculated by

(21) $$\Phi =\sqrt{\frac{1}{N}\sum_{i\in V}{\left(\alpha_{i,k|k}-\frac{1}{N}\sum_{j\in V}\alpha_{j,k|k}\right)}^{2}}.$$

The data in Figure 4 and Figure 5 are collated in Table 2.

The SD reduction of Algorithm 6 is the percentage by which Algorithm 6 reduces the SD compared to other algorithms. The convergence time of SD is the earliest time when SD reaches a steady state. From Table 1, we see that Algorithm 6 reduces the SD of estimation by up to 22.34% and reaches a steady state faster than CE-DKF and DHIF. This means that Algorithm 6 can ensure that all nodes in the sensor network have a higher degree of consensus. In the case of a small *Q*, Algorithm 6 only reduces SD by 0.81% compared with CE-DKF. The reason for this is the same as above: the error advantage of Algorithm 6 is small in the case of a small *Q*.

In each communication, each sensor applying Algorithm 6 only needs to send its own estimation $\hat{x}\left(t|t\right)\in R^{n}$ and the estimation error covariance $P\in R^{n\times n}$. Therefore, the communication traffic is n×n+n in terms of the number of transmitted digits. We give the communication bandwidth and frequency requirement of some popular algorithms in Table 3. The communication frequency in this study means the number of communications with an adjacent node required for each node to complete estimation. In addition to CE-DKF and DHIF, we compare the communication requirements of other two consensus-based algorithms. From Table 3, we can see that the communication bandwidth and frequency requirement of Algorithm 6 is the lowest (or one of the lowest).

## 4. Discussion

In this section, we discuss the result of Algorithm 6, including its estimation accuracy, degree of consensus, communication requirement, applicability to different topologies and robustness to local unobservability.

### 4.1. Estimation Accuracy

The reason why Algorithm 6 has better estimation accuracy than other diffusion-based algorithms is that Algorithm 6 applies the non-repeated diffusion strategy, which eliminates repeated information and reduces redundancy. In addition, Algorithm 6 improves the efficiency of information exchange due to non-redundant information exchange, meaning that it can converge to a steady-state value faster than other diffusion-based algorithms. The accuracy advantage of Algorithm 6 is small in the case of a small *Q*; this means that Algorithm 6 is good at estimating unknown models and maneuvering states. The only disadvantage is that Algorithm 6 compresses the fusion weight in the process of communication, which causes the fusion weight of Algorithm 6 to deviate and reduces the accuracy. This is a limitation of Algorithm 6. Future work will present a better weighting strategy.

### 4.2. Degree of Consensus

In sensor network estimation, it is very important that the estimation of all nodes is consistent. According to Section 3, Algorithm 6 has a lower estimation variance and a higher degree of consensus. It can be seen from Table 1 and Table 2 that the variance has a certain correlation with estimation error. Therefore, it can be considered that the reduction of error reduces the estimation dispersion range and the variance of Algorithm 6. Similarly, because the error converges faster, the consensus convergence rate is also faster. The advantage of the degree of consensus is small in the case of a small *Q*; this also means that Algorithm 6 is good at estimating unknown models and maneuvering states.

### 4.3. Communication Requirement

There are a large number of low-cost sensor networks with low communication bandwidths. For this kind of sensor network, the communication bandwidth requirement of the distributed filter algorithm has to be considered. From Section 3, we know that the communication traffic of Algorithm 6 is competitive against other distributed algorithms.

As with the communication bandwidth, communication frequency is limited for low-cost sensor networks. Some algorithms must satisfy a certain number of communications in order to receive global information and maintain a certain accuracy. When the number of communications is 1 Hz, Algorithm 6 can still guarantee that every node obtains global information.

When the communication frequency is less than 1 Hz, Algorithm 6 can still guarantee that every node obtains global information by combining measurements. In short, at a low communication frequency, as long as the communication frequency is greater than 0 Hz, Algorithm 6 can obtain better accuracy than some algorithms that must run in a certain communication frequency. This communication frequency requirement is also the advantage of diffusion-based algorithms, and thus we apply the diffusion-based algorithm and optimize it.

### 4.4. Applicability to Different Topologies

Theorem 2 assumes that the topology of the sensor network is an acyclic graph (tree graph). In fact, Algorithm 6 can also be applied to cyclic graphs. For sensor networks with a cyclic path, we can apply the spanning tree algorithm to convert a cyclic graph to an acyclic graph before applying Algorithm 6. Without doubt, this increases the complexity of Algorithm 6, which is also one of the limitations of Algorithm 6. Next, we can study this aspect and time-varying sensor networks.

### 4.5. Robustness to Local Unobservability

In some cases, some nodes miss the target but other nodes detect the target. The weight $w_{j\to i}$ of the nodes that miss the target can be considered to be 0. At this moment, Assumption 1 still holds. Since Theorem 2 is independent of the node weight, Algorithm 6 still guarantees that Theorem 2 holds. Therefore, Algorithm 6 is robust to local unobservability and is suitable for wider sensor networks.

## 5. Conclusions

In this study, a distributed Kalman filter is proposed based on the non-repetition diffusion strategy. It operates in a fully distributed mode and does not need to obtain information about the sensor network, which gives it excellent expansibility. The core of the algorithm lies in the non-repeated diffusion strategy, which optimizes the diffusion-based distributed Kalman filtering algorithms. Compared with existing distributed Kalman filtering algorithms, the algorithm proposed in this study has a lower estimation error. Besides, the algorithm also shows outstanding performance in terms of the degree of consensus, communication bandwidth requirement, communication frequency requirement, applicability to different topologies and robustness to local unobservability. The algorithm shows excellent performance in the tracking of maneuvering targets and the state estimation of low-cost sensor networks. By the simulation of a single target trace, we verify the performance of the algorithm. Future research topics include the weighting strategy and time-varying sensor networks.

## Figures and Tables

**Table 1:** Error performance comparison of different algorithms.

Algorithms	Steady State Error (m)		Error Reduction of Algorithm 6 (%)		Error Convergence Time (s)	
	$Q_{1}$	$Q_{2}$	$Q_{1}$	$Q_{2}$	$Q_{1}$	$Q_{2}$
Algorithm 6	0.7301	0.7297	0	0	7	7
CE-DKF	0.7476	0.8640	2.34	15.54	9	10
DHIF	0.9214	0.9233	20.76	20.97	8	9

**Table 2:** Consensus performance comparison of different algorithms.

Algorithms	Steady State Error (m)		SD Reduction of Algorithm 6 (%)		Convergence Time of SD (s)	
	$Q_{1}$	$Q_{2}$	$Q_{1}$	$Q_{2}$	$Q_{1}$	$Q_{2}$
Algorithm 6	0.6655	0.6702	0	0	7	7
CE-DKF	0.6710	0.7882	0.81	14.97	10	9
DHIF	0.8570	0.8631	22.34	22.35	8	8

**Table 3:** Communication requirements of different algorithms. FT-DKF: finite-time distributed Kalman filter; ICF: information consensus filter.

Algorithms	Bandwidth Requirement	Frequency Requirement (Hz)
Algorithm 6	n×n+n	1
ICF [13]	n×n+n	*d*
FT-DKF [18]	n×n+n	*d*
CE-DKF	n×n+n	1
DHIF	2×(n×n+n)	1

## Abbreviations

The following abbreviations are used in this manuscript:

ICF	Information consensus filter
FT-DKF	Finite-time distributed Kalman filter
CE-DKF	Cost-effective diffusion Kalman filter
CI	Covariance intersection
DHIF	Distributed hybrid information fusion
PDKF	Partial diffusion Kalman filter
EI	Ellipsoidal intersection
SD	Standard deviation
